# Proximal relationships between PTSD and drinking behavior

**DOI:** 10.3402/ejpt.v5.26518

**Published:** 2014-12-09

**Authors:** Debra Kaysen, Cynthia Stappenbeck, Issac Rhew, Tracy Simpson

**Affiliations:** Department of Psychiatry & Behavioral Sciences (PBSCI), University of Washington, Seattle, WA, USA

**Keywords:** alcohol abuse, drinking behavior, social support, sexual trauma, victimization, self-efficacy, comorbidity

## Abstract

Co-morbid PTSD and alcohol use disorders are both common and debilitating. However, many of these studies rely on cross-sectional studies that obscure more complex relationships between PTSD and drinking. Event-level studies allow for examination of proximal relationships between PTSD and drinking. Among women (*n*=136 with past sexual victimization, *n*=40 no past trauma history), a two-part mixed hurdle model was used to examine daily PTSD and drinking. On days women experienced more intrusive and behavioral avoidance symptoms, they were more likely to drink. For a 2 SD increase in symptoms, there was a 5% increased likelihood of drinking, and for a 2 SD increase in dysphoric symptoms or negative affect, women drank approximately half drink less. Daily-level coping self-efficacy moderated the association between distress and drinking (IRR=0.91, *p*<0.01). Women who reported less coping drank more as their distress increased on a certain day whereas women who reported more coping drank about the same regardless of distress. Overall, findings suggest that specific PTSD symptoms are associated with higher alcohol use and that these relationships are moderated by daily coping self-efficacy. Implications of these findings for informing models of PTSD/AUD comorbidity, as well as clinical implications will be discussed.

